# Generation and Validation of Normative, Age-Specific Reference Curves for Bone Strain Index in Women

**DOI:** 10.3390/diagnostics14101046

**Published:** 2024-05-18

**Authors:** Luca Rinaudo, Sofia Cuttone, Carmelo Messina, Veronica Magni, Davide Capra, Luca Maria Sconfienza, Francesco Sardanelli, Fabio Massimo Ulivieri

**Affiliations:** 1 Tecnologie Avanzate T.A. srl, Lungo Dora Voghera 36/A, 10153 Torino, Italy; 2 IRCCS Istituto Ortopedico Galeazzi, 20157 Milano, Italy; 3 Dipartimento di Scienze Biomediche per la Salute, Università degli Studi di Milano, 20122 Milano, Italy; 4 Postgraduate School in Radiodiagnostics, Università degli Studi di Milano, 20122 Milano, Italy; 5 IRCCS Policlinico San Donato, Via Morandi 30, 20097 San Donato Milanese, Italy; 6 Fondazione IRCCS Ca’Granda Ospedale Maggiore Policlinico, Via F. Sforza 35, 20122 Milano, Italy

**Keywords:** L-BSI, F-BSI, Bone Strain Index (BSI), DXA, normative data, distribution

## Abstract

Bone Strain Index (BSI), based on dual-energy X-ray absorptiometry (DXA), is a densitometric index of bone strength of the femur and lumbar spine. Higher BSI values indicate a higher strain applied to bone, predisposing to higher fracture risk. This retrospective, multicentric study on Italian women reports the BSI normative age-specific reference curves. A cohort of Caucasian Italian women aged 20 to 90 years was selected from three different clinical centres. Bone mineral density (BMD) and BSI measurements were obtained for the lumbar spine vertebrae (L1–L4) and for the femur (neck, trochanter and intertrochanter) using Hologic densitometers scans. The data were compared with BMD normative values provided by the densitometer manufacturer. Then, the age-specific BSI curve for the femur and lumbar spine was generated. No significant difference was found between the BMD of the subjects in this study and BMD reference data provided by Hologic (*p* = 0.68 for femur and *p* = 0.90 for lumbar spine). Spine BSI values (L1–L4) increase by 84% between 20 and 90 years of age. The mean BSI of the total femur increases about 38% in the same age range. The BSI age-specific reference curve could help clinicians improve osteoporosis patient management, allowing an appropriate patient classification according to the bone resistance to the applied loads and fragility fracture risk assessment.

## 1. Introduction

Osteoporosis represents a skeletal disorder characterised by reduced bone strength that leads to an increased fracture risk [1,2]. Bone strength is mainly determined by bone mineral density (BMD), bone micro-architecture and the ability of bone to deform under loads [3]. The quantitative evaluation of BMD, based on dual-energy X-ray absorptiometry (DXA), is the reference standard in clinical routine [4]. It has been shown that the risk of fracture doubles for one standard deviation reduction in BMD measured using DXA, as density and failure of a loaded material have a quadratic relationship [3,5]. However, BMD alone may lack sensitivity for individual fracture risk assessment, as many patients presenting with an incident or prevalent fragility fracture show BMD values in the osteopenic range [6]. Indeed, BMD cannot evaluate the above-mentioned bone strength determinants: bone texture and bone deformation capability [3,7]. Other radiological indexes based on DXA have been proposed to enhance fracture prediction. Trabecular Bone Score (TBS), available since 2008, is a DXA-derived index of bone texture that shows a reliable relationship with bone histomorphometric parameters [8,9] and a specific ability to predict fracture risk [10,11,12]. Nonetheless, TBS is performed only on one skeletal site and does not provide information about the capability of bone to deform under loads, a feature that affects—like all materials—a structure’s resistance to load [3,13]. A novel DXA-based bone parameter has been developed since 2018, the Bone Strain Index (BSI). BSI represents a bone deformation index based on Finite Element Analysis of a lumbar spine (L-BSI) and a proximal femoral (F-BSI) DXA scan. BSI can be automatically calculated from DXA examinations [3,14].

In primary osteoporosis, L-BSI and F-BSI demonstrate the ability to predict all fragility fractures [15]. L-BSI is useful for the identification of osteoporotic female subgroups with a particular tendency to first fragility fractures [16] and to successive fractures [17,18]. Furthermore, low values of F-BSI seem to be connected with the non-occurrence of vertebral fractures [19]. In secondary osteoporosis, L-BSI demonstrated good ability in discriminating vertebral fractured patients affected by hyperparathyroidism [20,21] and appeared to be useful in the clinical characterisation of patients affected by mastocytosis [22] and recessive dystrophic epidermolysis bullosa [23], whereas F-BSI proved to be associated with vertebral fractures in aromatase inhibitor naive patients [24]. In addition, BSI was able to detect patients treated with anabolic osteoporotic that do not present BMD increase [25].

As for BMD and TBS, normal reference data for BSI are needed to improve its clinical use. A preliminary distributional characteristic of women with osteoporosis, osteopenia and normal BMD has been recently published, indicating in women without vertebral fractures, a BSI value lower than 1.68 for the non-osteoporotic group and higher than 2.40 for the non-normal group [26]. However, the previous work [26] considered a limited study sample size, which was not satisfactory for evaluating the distributional characteristics of a quantitative variable. In addition, the reported data are limited to women without fractures at baseline [26]. In this work, an “all-comers approach” allows us to analyse a larger cohort, regardless of health conditions.

The present study aims to present BSI normative age-specific data in a large, retrospective, multicentric cohort of Caucasian women.

## 2. Materials and Methods

### 2.1. Study Population

The study population was derived from databases of outpatients performing osteoporosis DXA evaluation at three different clinical sites [26]. Study subjects were selected from patient lists at three different Italian hospitals: IRCCS Istituto Ortopedico Galeazzi, Milan; IRCCS Policlinico San Donato, San Donato Milanese; Fondazione IRCCS Ca’ Grande Ospedale Maggiore Policlinico of Milan.

Local ethical committee approvals were obtained by the respective ethical committees of the involved hospitals (Comitato Etico Milano Area 2. Protocol N 2.0 BQ. 265_2017, 13 June 2017; Comitato Etico San Raffaele; Studio clinico 2.0 BQ, version 4.0, 8 August 2019). Patients included in the present study provided written consent for anonymised data usage for research purposes at the moment of DXA examination [26].

From these three centres, a cohort of Caucasian female participants was selected, aged 6 to 90 years, who had a DXA exam on the femur or lumbar spine or both sites. The initial cohort of white female subjects included a total of 17,563 femoral and 16,721 lumbar DXA examinations. An “all-comers approach” has been used for this study to consider a more representative group of the overall population [27]. This approach enrols subjects regardless of their health condition, as a large number of samples can be considered more representative of the overall population. Therefore, the “all-comers approach” incorporates within the study population subjects with fractures, under treatment or with various bone disorders [27]. Data about comorbidities or treatment were obtained using a specific anamnestic questionnaire provided before the DXA examination.

**Figure 1:**
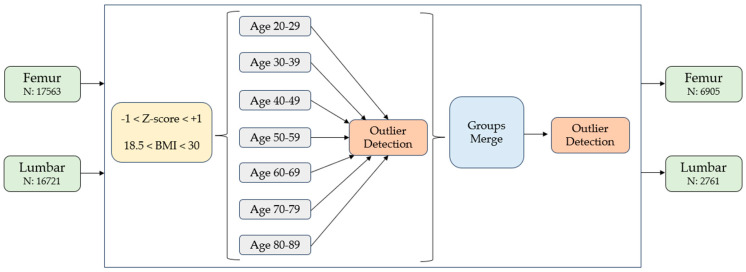
Graphic summary of the methodology: (1) “all-comers approach” was taken to enrol patients in the study; (2) only subjects with −1 < Z-score < 1 and 18.5 kg/m^2^ <BMI < 30 kg/m^2^ were selected; (3) the population was divided into 7 age groups; (4) for each age group, an outlier detection process was used; (5) all age groups were merged into one group; (6) the outlier detection process was used again. At the end of the process, 6905 hip scans and 2761 spine scans were selected. The BMD and BSI curves were constructed on these.

In order to obtain a cohort of patients with normal BMD values in relation to age and sex, only subjects with −1 < Z-score < 1 were selected. Furthermore, subjects with BMI < 18.5 kg/m^2^ and BMI > 30 kg/m^2^ were excluded to obtain a normal weight population. Participants younger than 20 years were also excluded, and the population was divided into 7 age groups: 20–29, 30–39, 40–49, 50–59, 60–69, 70–79, 80–89. After an outlier detection process, 6905 women for the femur and 2761 for the lumbar spine were included for the construction of the age-specific BSI curves. This methodology is graphic in Figure 1.

### 2.2. BMD and BSI Measurements

BMD evaluation was performed with DXA using a Hologic QDR-Discovery W (IRCCS Istituto Ortopedico Galeazzi), a Hologic Delphi (IRCCS Policlinico San Donato), and a Hologic Discovery A (Fondazione IRCCS Ca’ Grande Ospedale Maggiore Policlinico of Milan and IRCCS Policlinico San Donato). Lumbar spine BMD measurements were obtained for the four lumbar spine vertebrae (L1–L4); femur BMD assessment was performed for the neck, trochanter, intertrochanter and total hip. All DXA scans were acquired according to the International Society for Clinical Densitometry official positions and performed by trained technicians.

**Figure 2:**
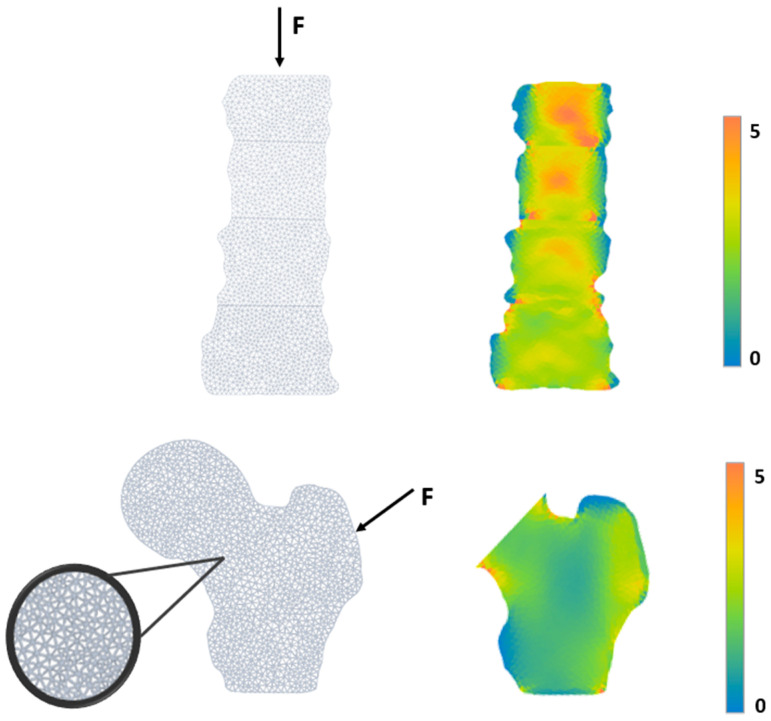
Examples of lumbar and femur BSI analysis: The BSI calculation uses Finite Element Method (FEM). The FEM analysis generates a 2D model of the bone segment starting from the bone segmentation, carried out by the DXA software (v5.6). A patient-specific load is applied to each 2D model, divided into triangular elements (mesh). Based on the density distribution, the bone strain is calculated at each geometric element of the mesh. BSI represents the average equivalent strain inside the bone, with the assumption that a higher strain level (high BSI) indicates a more significant risk condition [3,14].

Bone Strain Index Software (v1.4.0, Tecnologie Avanzate T.A. s.r.l., Turin, Italy) was used to produce a finite element analysis of lumbar spine and femur DXA scans and calculate BSI values. BSI was calculated on the same region of interest of the DXA area as the average equivalent strain for each vertebra and for each femoral region. Finally, the mean BSI of the total femur and total lumbar spine was evaluated. Figure 2 shows the representation of the Bone Strain Index calculation from lumbar and hip DXA scans. This process is automatically performed by the BSI software that creates a report with BSI values immediately after the end of DXA analysis.

### 2.3. Construction of the Database

For each subject, the following parameters were extracted from DXA exams and from BSI software: patient age, weight, height, BMI, BMD and BSI for each vertebra alone, for the total lumbar spine, for each femur region and for the total femur. Data from all subjects were anonymised before including them in the study and then saved in an Excel file.

### 2.4. Age-Specific BSI Curve Creation

MATLAB (v2021a, MathWorks Inc, Natick, MA, USA) was used to create age-related BSI curves, and the process consisted of three steps: (1) dataset creation starting from the database available, (2) dataset validation, and (3) curves generation [27].

#### 2.4.1. Dataset Creation

For each age group, an outlier detection process was used for height, weight, BMI, BMD and BSI parameters for each vertebra, for all four vertebrae together, for each femoral region and for the total femur.

A “box and whisker outlier method” was used to consider as “outliers” those variables out of 1.5 interquartile ranges above the upper quartile (75%) or below the lower quartile (25%). These outliers were excluded until no others were detected for any study parameter. Then, values from all age groups were merged into one group, with further application of the outlier detection process until no outliers were finally found for any parameter for the overall subject group.

#### 2.4.2. Dataset Validation

Age-related BMD curves provided by the manufacturer of the densitometer (Hologic Inc., Marlborough, MA, USA) were used as a reference to evaluate the normality of the dataset. Based on the created dataset, age-related BMD curves for white women were constructed and then compared with reference Hologic curves. The mean BMDs of the study sample and the reference ones were compared by calculating relative error and by performing the Student’s t-test. The relative error was considered acceptable if less than 5%. The final dataset was deemed to be representative of the normal population if no significant differences existed between the generated age-related BMD curve and the referenced BMD Curve. Any *p*-value greater than 0.05 was considered statistically insignificant.

#### 2.4.3. BSI Curves Generation

From the validated dataset, the age-specific BSI curve for the femur and lumbar spine was generated with linear regression. Mean and standard deviation were calculated for each age group. An age-specific curve was constructed for each vertebra and for all vertebrae together, for each femur region and for the total femur.

### 2.5. Statistical Analysis

Statistical analysis was performed with the use of Statgraphics (v.18, Statgraphics Technologies Inc., The Plains, VA, USA). Descriptive statistics were obtained to provide an overview of the characteristics of the study population for each age group. Additionally, the enrolled women were divided into three groups based on their bone mass: women with osteoporosis, women with osteopenia, and women with normal bone mass. The mean values of Age, BMI, BMD and BSI ± SD and the median ranges with the first and third quartiles were determined. Parametric (P) and non-parametric (NP) 95% confidence intervals (CI) were calculated for the generation of the BSI reference limits [26].

## 3. Results

### 3.1. Descriptive Statistics for the Study Samples

After the outlier detection process, 6905 women for the femur and 2761 for the lumbar spine were selected for the construction of the age-specific BSI curves.

Based on the lumbar T-Score values, the percentages of the study population defined as osteoporotic, osteopenic, and normal were 16.3%, 62.4%, and 21.4%, respectively. Regarding the proximal femur, 4% of women enrolled in the present study presented T-score values within the range of osteoporosis, 66% were osteopenic, and 30% showed normal values. Table 1 and Table 2 illustrate the baseline features of the three groups of women with osteoporosis, osteopenia and normal bone mass for lumbar and femoral scans, respectively.

#### 3.1.1. Cohort for Femur

Subjects were subdivided into seven age groups, including 56 women between the ages of 20 and 29, inclusive; 79 women, 30–39; 391 women, 40–49; 1518 women, 50–59; 2474 women, 60–69; 1952 women, 70–79; and 392 women, 80–89. The mean BMD values for the total femur, mostly constant from 20 to 50 years of age, undergo a 19% decrease, about 4% per ten years, between 40 and 90 years of age. Height decreases with increasing age, with almost the same trend of BMD.

#### 3.1.2. Cohort for Lumbar Spine

Subjects were subdivided into seven age groups, including 30 women between the ages of 20 and 29, inclusive; 68 women, 30–39; 303 women, 40–49; 776 women, 50–59; 959 women, 60–69; 538 women, 70–79; and 81 women, 80–89. The mean BMD values for the lumbar spine (L1–L4) decrease by about 5% between the ages of 20 and 50 years of age, by 20% between 50 and 90 years, and by about 5% per 10 years. As for the femur, height and BMD follow an inverse relationship with age, according to the trend described by the Hologic reference curves [28].

### 3.2. Age-Specific BMD Reference Data Comparison

The relative error between the mean BMD of the study sample and the reference one was found to be acceptable (3.6% for the total femur and 1.9% for the lumbar spine).

We found no significant difference in BMD values between subjects enrolled in the current study (BMDstd) and those from the BMD reference data (BMDref) provided by Hologic for white women (FemurBMDref = 0.83 ± 0.12 g/cm^2^ vs. FemurBMDstd = 0.80 ± 0.05 g/cm^2^; *p* = 0.68; LumbarBMDref = 0.90 ± 0.11 g/cm^2^ vs. LumbarBMDstd = 0.89 ± 0.04 g/cm^2^; *p* = 0.90).

### 3.3. BSI Age-Related Changes

#### 3.3.1. Lumbar Spine

Spine BSI values increase with age and with decreasing BMD, irrespective of the region of interest (ROI) chosen (Table 1).

The mean BSI of the lumbar spine (L1–L4) increased by 84% between 20 and 90 years of age. The increase is not constant over the years: it is equal to 2% between 20 and 40 years of age, after the age of 40 it increases sharply (about 20% every 10 years) to 80 years, then it decreases again to 2% between 80 and 90 years of age.

The curve can be visually divided into three areas with different slopes: before 35 years, between 35 and 75 years, and after 75 years. Age-related BSI values follow a similar trend in individual vertebrae and in the total lumbar spine (L1–L4): between 20 and 35 years, the curve increases slowly, to then undergo a sudden increase between 35 and 75 years; after 75 years, the BSI values continue to increase with a lower slope and very similar to that between 20 and 35 years. In particular, in L1–L4, the segment between 35 and 75 years has a slope approximately 7.5 times higher than the segment between 20 and 35 years and 0.10 times lower than the next segment, between 75 and 90 years. Full data is reported in Table 3.

**Figure 3:**
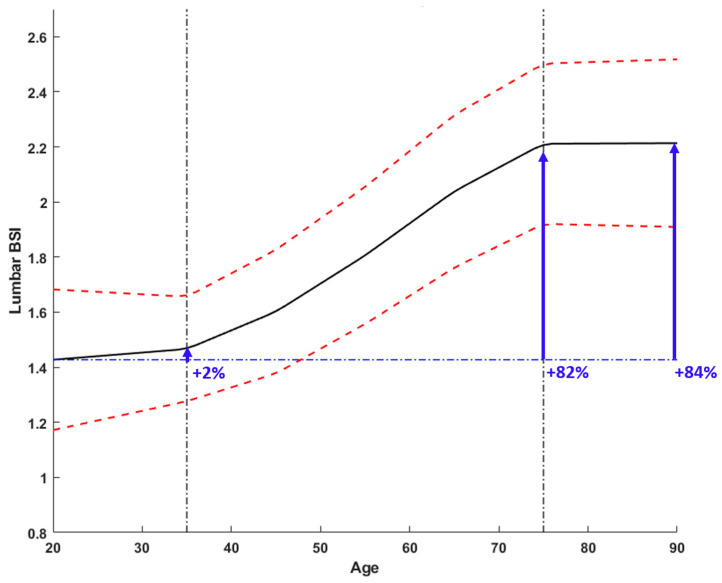
BSI age-related changes for lumbar spine (L1–L4). The mean BSI value for the specific age is represented by the black. Red dashed lines represent the two standard deviation lines. The graph highlights three different areas depending on the slope: before 35 years, between 35 and 75 years, and after 75 years. BSI value increases by 2% between 20 and 35 years (slope equal to 0.0026/year), by 78% between 35 and 75 years (slope equal to 0.0193/year), and by 2% after 75 years (slope equal to 0.0021/year).

These results are depicted in Figure 3.

#### 3.3.2. Femur

F-BSI values increase with age in any ROI. This increase is less abrupt when compared with L-BSI. Indeed, the mean BSI of the total femur increases by about 15% between 20 and 60 years of age and 23% between 60 and 90 years of age, totalling about 38% between 20 and 90 years of age. Comparing these data with those of the previous paragraph, it emerges that the L-BSI has increased over the years by 2.2 times the F-BSI.

**Figure 4:**
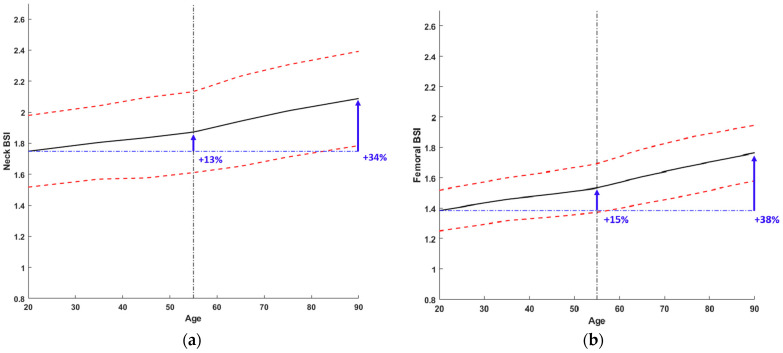
BSI age-related changes in the femur. (**a**) Reference curve for Femoral Neck and (**b**) for Total Femur. The black line represents the mean BSI value for age. The red dashed lines represent the two SD lines. The curve shows a little slope change before 55 years and after 55 years.

As shown in Figure 4, two different increases with age can be observed regardless of the ROI chosen: before age 55 and after age 55. After age 55, Neck BSI and F-BSI values increased at a rate three times and 2.7 times faster, respectively, than before age 55. Full data is reported in Table 4.

#### 3.3.3. Confidence Limits

Table 5 summarises the parametric and nonparametric 95% confidence limits L-BSI values (95% CI.P and 95% CI.NP) in the three groups of women with osteoporosis, osteopenia and normal bone mass. The nonparametric 95% confidence limits were similar to those obtained in [26].

## 4. Discussion

This is the first multicentric retrospective study that generates age-specific reference values for lumbar and femoral BSI of Caucasian Italian women aged 20–90. The average BMI in our population is 23.9 kg/m^2^, a value that aligns closely with the average BMI reported in other studies conducted on Western populations [29].

High BSI means a condition of worse resistance of bone to loads [3]. Lumbar and femoral BSI increases with age with different slopes of the curve, according to the trend of the decrease in BMD. This is an expected behaviour, given the dependence of BSI calculation from BMD values. This age-related fracture resistance loss is quite similar to what occurs with areal BMD in the lumbar spine (L1–L4), decreasing most rapidly between 45 and 65 years of age (about 0.010 g/cm^2^ per year), with a slower decrease rate thereafter (approximately 0.002 g/cm^2^ per year) [27]. In fact, BSI calculation considers the patient’s BMD, weight, and the shape of the skeletal site where BSI is measured, and BMD is the variable with a greater impact in the calculation [3,14].

In many studies focusing on age-specific curve construction, a controlled study design with specified inclusion and exclusion criteria was used. This method has the advantage of including in the study only patients who meet certain defined criteria, for example, only healthy patients. However, it is rigid and allows you to enrol a small cohort of subjects. In this study, to create our age-specific BSI curve, we have chosen an alternative method which has already been validated for the TBS normative curve [27], also known as the “all-comers approach”. This approach, frequently used in clinical research for biomarker evaluations, avoids the question of defining “healthy subjects” and considers the much larger quantity of subjects enrolled (all-comers) far more representative of the overall population. A flaw of this approach is that it incorporates within the study population people with fractures, those suffering from diseases that could affect bone, and those on pharmacological treatment that could impact bone metabolism. To avoid this bias, a cohort validation process was developed. First, more than the usual sample size used for conventional age-specific curve construction was included. Then, despite we did not use inclusion or exclusion criteria, we adopted a box and whisker outlier exclusion method (which means that we excluded from this study those with atypical height, weight, BMI, BMD, and Z-score values). Finally, to ensure the representativeness of our sample, we conducted a comparison between our age-specific BMD curve and an age-specific BMD curve for Caucasian women that was supplied by Hologic for their bone densitometers; this analysis revealed no significant differences between the two curves. So, we can assume that our study population is effectively representative of normal Italian Caucasian women for what concerns their BMD with age curve.

Following a previous preliminary study [26], this new study, based on a larger cohort, validates the normative BSI values, providing a new reference: lumbar BSI values equal to 1.78 and 2.05 can be used to differentiate the bone stress conditions typical of normal patients from those of osteoporotic patients.

Looking at Figure 3, these values seem to be reasonable since the lower threshold identifies a patient group with BSI typical of young females, whereas the higher threshold identifies a patient group with a BSI value higher than that of post-menopausal women. Using the same considerations for the femoral site, it is possible to identify a normal range for F-BSI < 1.56 and Neck BSI < 1.74 and a major risk condition for F-BSI > 1.79 and Neck BSI > 2.32 since corresponding to a not normal population. In addition, it is worth mentioning that the age range is very wide (20–90 years), like that considered for the BMD curve, and in contrast with other normative age-related DXA bone quality parameter curves [27].

This age-related reference curve will help clinicians to better interpret BSI results for their individual patients, but some limitations must be taken into consideration.

The “all-comers approach” applied to our study included different potentially confounding conditions (such as the presence of vertebral fracture or degenerative changes, possible treatments and concomitant diseases affecting bone). Consequently, the study population cannot be considered entirely healthy because of the presence of possible diseases and bone-impacting drugs. Furthermore, although the dataset used is very wide, the age groups between 20–40 and 80–90 are less represented than the others. This is intrinsically linked to the DXA exam, which, according to WHO indications, is indicated in 65-year-old women and 70-year-old men or, in the case of major risk factors for osteoporosis, in post-menopausal women or in women with transitional menopause as well as in men even under the age of 70 [30].

A third limitation is that this normative age-related curve deals only with females, and males are not considered. Certainly, osteoporosis also affects men, but it is the female sex that is more involved in the age 45–65 years, where post-menopausal osteoporosis can impair bone mass and affect the future health status of the woman. Therefore, we felt it was more urgent to devote ourselves to the elaboration of a trend curve of BSI for women before men.

Finally, this study carries the limitations of its retrospective nature, but these are limited considering the purpose of developing reference curves, which can be used for future prospective studies.

Although BMD is the most influential variable in the BSI calculation, the patient’s weight and skeletal site shape also influence the BSI values. In fact, from a physical point of view, the strength of an object under load depends on the density per volume of the construction material of the object, the spatial distribution of the material and the capability to deform under load on the object [3]. In bone densitometry, the first is represented by BMD, the second by trabecular bone score (TBS), and the third by BSI [3]. BSI provides, for this reason, information which, in combination with the others, completes the patient’s clinical profile, improving the prediction of fragility fracture risk in subjects classified as osteopenic/osteoporotic based on BMD alone. The importance of the BSI reference curves for a better interpretation of the results is, therefore, evident.

## 5. Conclusions

We present age-specific reference curves for lumbar and femoral BSI derived from a multicentre database encompassing 6905 women for the femur and 2761 for the lumbar spine, spanning ages 20 to 90 years. Despite inherent limitations associated with the retrospective “all-comers approach,” the provided curves and value ranges can aid clinicians in enhancing fracture risk assessment and the management of osteoporosis patients by allowing appropriate patient classification based on bone resistance to applied loads.

## Figures and Tables

**Table 1:** Baseline characteristics of the enrolled women were grouped according to their bone mineral density for Lumbar Spine.

	Osteoporosis Mean (±SD) Median (Q1–Q3)	Osteopenia Mean (±SD) Median (Q1–Q3)	Normal Mean (±SD) Median (Q1–Q3)
Lumbar (Number)	449	1722	590
Age (Year)	74.4 (5.50) 74 (70–78)	63.5 (7.34) 64 (58–68)	47.7 (8.45) 49 (44–53)
BMI	23.85 (2.60) 23.79 (21.88–25.78)	24.10 (2.83) 23.83 (21.97–26.27)	23.84 (2.91) 23.60 (21.48–26.03)
Lumbar BMD	0.746 (0.024) 0.752 (0.732–0.766)	0.850 (0.042) 0.849 (0.814–0.885)	0.981 (0.035) 0.976 (0.952–1.003)
Lumbar BSI	2.288 (0.258) 2.286 (2.101–2.484)	1.972 (0.279) 1.956 (1.774–2.158)	1.598 (0.233) 1.604 (1.426–1.77)

**Table 2:** Baseline characteristics of the enrolled women grouped according to their bone mineral density for the Proximal Femur.

	Osteoporosis Mean (±SD) Median (Q1–Q3)	Osteopenia Mean (±SD) Median (Q1–Q3)	Normal Mean (±SD) Median (Q1–Q3)
Femur (Number)	449	1722	590
Age (Year)	213	4558	2134
BMI	79.6 (4.50) 79 (76–83)	68.9 (8.04) 69 (63–74)	56.5 (10.06) 57 (51–64)
Femoral BMD	22.85 (2.61) 22.37 (20.81–24.67)	24.28 (2.65) 24.16 (22.31–26.27)	24.80 (2.77) 24.83 (22.62–26.99)
Femoral BSI	0.623 (0.016) 0.628 (0.614–0.636)	0.744 (0.043) 0.747 (0.713–0.779)	0.865 (0.038) 0.857 (0.833–0.891)
Neck BMD	1.883 (0.146) 1.896 (1.778–2.001)	1.655 (0.172) 1.649 (1.531–1.772)	1.477 (0.146) 1.471 (1.374–1.575)
Neck BSI	0.542 (0.044) 0.537 (0.511–0.569)	0.621 (0.052) 0.62 (0.584–0.655)	0.718 (0.055) 0.715 (0.68–0.758)

**Table 3:** Mean Lumbar-BSI ± Standard Deviation for each age group and ROI.

Age Group (y)	L1	L2	L3	L4	Total
20–29	1.65 ± 0.27	1.46 ± 0.23	1.36 ± 0.24	1.29 ± 0.23	1.44 ± 0.23
30–39	1.67 ± 0.23	1.49 ± 0.18	1.38 ± 0.18	1.33 ± 0.20	1.47 ± 0.19
40–49	1.83 ± 0.26	1.62 ± 0.24	1.52 ± 0.22	1.45 ± 0.22	1.61 ± 0.23
50–59	2.05 ± 0.28	1.83 ± 0.26	1.72 ± 0.25	1.63 ± 0.26	1.81 ± 0.25
60–69	2.30 ± 0.31	2.06 ± 0.29	1.94 ± 0.28	1.83 ± 0.28	2.03 ± 0.28
70–79	2.50 ± 0.33	2.21 ± 0.30	2.11 ± 0.29	2.00 ± 0.30	2.20 ± 0.29
80–89	2.49 ± 0.36	2.23 ± 0.31	2.12 ± 0.30	2.04 ± 0.29	2.22 ± 0.30

**Table 4:** Mean Femur-BSI ± Standard Deviation for each age group and ROI.

Age Group (y)	Neck	Inter	Troch	Total
20–29	1.77 ± 0.23	1.14 ± 0.14	1.33 ± 0.12	1.41 ± 0.14
30–39	1.81 ± 0.23	1.18 ± 0.16	1.37 ± 0.13	1.45 ± 0.16
40–49	1.84 ± 0.24	1.20 ± 0.15	1.44 ± 0.14	1.50 ± 0.14
50–59	1.87 ± 0.26	1.22 ± 0.16	1.51 ± 0.16	1.53 ± 0.16
60–69	1.94 ± 0.29	1.27 ± 0.17	1.61 ± 0.18	1.61 ± 0.18
70–79	2.01 ± 0.30	1.32 ± 0.17	1.69 ± 0.19	1.67 ± 0.19
80–89	2.06 ± 0.30	1.35 ± 0.17	1.78 ± 0.18	1.73 ± 0.18

**Table 5:** Summary of parametric and nonparametric 95% confidence limits Lumbar-BSI values (95%CI.P and 95%CI.NP) in the three groups of women with osteoporosis, osteopenia and normal bone mass.

	Osteoporosis	Osteopenia	Normale
Lumbar BSI			
95% CI.P	1.796; 2.758	1.425; 2.519	1.141; 2.055
95% CI.NP	1.782; 2.794	1.473; 2.557	1.158; 2.05
Femur BSI			
95% CI.P	1.597; 2.169	1.318; 1.992	1.191; 1.763
95% CI.NP	1.562; 2.112	1.34; 1.998	1.205; 1.778
Neck BSI			
95% CI.P	1.748; 2.783	1.436; 2.546	1.32; 2.3
95% CI.NP	1.744; 2.694	1.459; 2.549	1.336; 2.317

## Data Availability

The data presented in this study are available on request from the corresponding author due to legal reasons.

## References

[1] WA P. (1993). Consensus Development Conference: Diagnosis, Prophylaxis, and Treatment of Osteoporosis. Am. J. Med..

[2] Kanis J.A., Cooper C., Rizzoli R., Reginster J.-Y. (2019). European Guidance for the Diagnosis and Management of Osteoporosis in Postmenopausal Women. Osteoporos. Int..

[3] Ulivieri F.M., Rinaudo L. (2021). Beyond Bone Mineral Density: A New Dual X-Ray Absorptiometry Index of Bone Strength to Predict Fragility Fractures, the Bone Strain Index. Front. Med..

[4] (1994). Assessment of Fracture Risk and Its Application to Screening for Postmenopausal Osteoporosis. Report of a WHO Study Group.

[5] Marshall D., Johnell O., Wedel H. (1996). Meta-Analysis of How Well Measures of Bone Mineral Density Predict Occurrence of Osteoporotic Fractures. BMJ.

[6] Siris E.S., Chen Y.-T., Abbott T.A., Barrett-Connor E., Miller P.D., Wehren L.E., Berger M.L. (2004). Bone Mineral Density Thresholds for Pharmacological Intervention to Prevent Fractures. Arch. Intern. Med..

[7] Seeman E., Delmas P.D. (2006). Bone Quality—The Material and Structural Basis of Bone Strength and Fragility. N. Engl. J. Med..

[8] Krohn K., Schwartz E.N., Chung Y.-S., Lewiecki E.M. (2019). Dual-Energy X-Ray Absorptiometry Monitoring with Trabecular Bone Score: 2019 ISCD Official Position. J. Clin. Densitom..

[9] Hans D., Barthe N., Boutroy S., Pothuaud L., Winzenrieth R., Krieg M.-A. (2011). Correlations Between Trabecular Bone Score, Measured Using Anteroposterior Dual-Energy X-Ray Absorptiometry Acquisition, and 3-Dimensional Parameters of Bone Microarchitecture: An Experimental Study on Human Cadaver Vertebrae. J. Clin. Densitom..

[10] Hans D., Goertzen A.L., Krieg M.-A., Leslie W.D. (2011). Bone Microarchitecture Assessed by TBS Predicts Osteoporotic Fractures Independent of Bone Density: The Manitoba Study. J. Bone Miner. Res..

[11] Pothuaud L., Barthe N., Krieg M.-A., Mehsen N., Carceller P., Hans D. (2009). Evaluation of the Potential Use of Trabecular Bone Score to Complement Bone Mineral Density in the Diagnosis of Osteoporosis: A Preliminary Spine BMD–Matched, Case-Control Study. J. Clin. Densitom..

[12] Silva B.C., Leslie W.D. (2017). Trabecular Bone Score. Endocrinol. Metab. Clin. N. Am..

[13] Mirzaali M.J., Libonati F., Ferrario D., Rinaudo L., Messina C., Ulivieri F.M., Cesana B.M., Strano M., Vergani L. (2018). Determinants of Bone Damage: An Ex-Vivo Study on Porcine Vertebrae. PLoS ONE.

[14] Ulivieri F.M., Rinaudo L. (2022). The Bone Strain Index: An Innovative Dual X-Ray Absorptiometry Bone Strength Index and Its Helpfulness in Clinical Medicine. J. Clin. Med..

[15] Sornay-Rendu E., Duboeuf F., Ulivieri F.M., Rinaudo L., Chapurlat R. (2022). The Bone Strain Index Predicts Fragility Fractures. The OFELY Study. Bone.

[16] Ulivieri F.M., Piodi L.P., Grossi E., Rinaudo L., Messina C., Tassi A.P., Filopanti M., Tirelli A., Sardanelli F. (2018). The Role of Carboxy-Terminal Cross-Linking Telopeptide of Type I Collagen, Dual x-Ray Absorptiometry Bone Strain and Romberg Test in a New Osteoporotic Fracture Risk Evaluation: A Proposal from an Observational Study. PLoS ONE.

[17] Ulivieri F.M., Piodi L.P., Rinaudo L., Scanagatta P., Cesana B.M. (2020). Bone Strain Index in the Prediction of Vertebral Fragility Refracture. Eur. Radiol. Exp..

[18] Messina C., Rinaudo L., Cesana B.M., Maresca D., Piodi L.P., Sconfienza L.M., Sardanelli F., Ulivieri F.M. (2021). Prediction of Osteoporotic Fragility Re-Fracture with Lumbar Spine DXA-Based Derived Bone Strain Index: A Multicenter Validation Study. Osteoporos. Int..

[19] Ulivieri F.M., Rinaudo L., Piodi L.P., Messina C., Sconfienza L.M., Sardanelli F., Guglielmi G., Grossi E. (2021). Bone Strain Index as a Predictor of Further Vertebral Fracture in Osteoporotic Women: An Artificial Intelligence-Based Analysis. PLoS ONE.

[20] Tabacco G., Naciu A.M., Messina C., Sanson G., Rinaudo L., Cesareo R., Falcone S., Napoli N., Ulivieri F.M., Palermo A. (2023). DXA-Based Bone Strain Index in Normocalcemic Primary Hyperparathyroidism. Osteoporos. Int..

[21] Tabacco G., Naciu A.M., Messina C., Sanson G., Rinaudo L., Cesareo R., Falcone S., Manfrini S., Napoli N., Bilezikian J.P. (2021). DXA-Based Bone Strain Index: A New Tool to Evaluate Bone Quality in Primary Hyperparathyroidism. J. Clin. Endocrinol. Metab..

[22] Ulivieri F.M., Rinaudo L., Piodi L.P., Barbieri V., Marotta G., Sciumè M., Grifoni F.I., Cesana B.M. (2020). Usefulness of Dual X-Ray Absorptiometry-Derived Bone Geometry and Structural Indexes in Mastocytosis. Calcif. Tissue Int..

[23] Rodari G., Guez S., Salera S., Ulivieri F.M., Tadini G., Brena M., Profka E., Giacchetti F., Arosio M., Giavoli C. (2022). A Single-Centre Study on Predictors and Determinants of Pubertal Delay and Growth Impairment in Epidermolysis Bullosa. PLoS ONE.

[24] Pedersini R., Cosentini D., Rinaudo L., Zamparini M., Ulivieri F.M., di Mauro P., Maffezzoni F., Monteverdi S., Vena W., Laini L. (2023). Assessment of DXA Derived Bone Quality Indexes and Bone Geometry Parameters in Early Breast Cancer Patients: A Single Center Cross-Sectional Study. Bone Rep..

[25] Messina C., Piodi L.P., Grossi E., Eller-Vainicher C., Bianchi M.L., Ortolani S., Di Stefano M., Rinaudo L., Sconfienza L.M., Ulivieri F.M. (2020). Artificial Neural Network Analysis of Bone Quality DXA Parameters Response to Teriparatide in Fractured Osteoporotic Patients. PLoS ONE.

[26] Ulivieri F.M., Rinaudo L., Messina C., Aliprandi A., Sconfienza L.M., Sardanelli F., Cesana B.M. (2022). Bone Strain Index: Preliminary Distributional Characteristics in a Population of Women with Normal Bone Mass, Osteopenia and Osteoporosis. Radiol. Med..

[27] Dufour R., Winzenrieth R., Heraud A., Hans D., Mehsen N. (2013). Generation and Validation of a Normative, Age-Specific Reference Curve for Lumbar Spine Trabecular Bone Score (TBS) in French Women. Osteoporos. Int..

[28] Looker A.C., Wahner H.W., Dunn W.L., Calvo M.S., Harris T.B., Heyse S.P., Johnston C.C., Lindsay R. (1998). Updated Data on Proximal Femur Bone Mineral Levels of US Adults. Osteoporos. Int..

[29] Nyberg S.T., Heikkilä K., Fransson E.I., Alfredsson L., De Bacquer D., Bjorner J.B., Bonenfant S., Borritz M., Burr H., Casini A. (2012). Job Strain in Relation to Body Mass Index: Pooled Analysis of 160 000 Adults from 13 Cohort Studies. J. Intern. Med..

[30] Rossini M., Adami S., Bertoldo F., Diacinti D., Gatti D., Giannini S., Giusti A., Malavolta N., Minisola S., Osella G. (2016). Guidelines for the Diagnosis, Prevention and Management of Osteoporosis. Reumatismo.

